# Exploring Patient Values in Medical Decision Making: A Qualitative Study

**DOI:** 10.1371/journal.pone.0080051

**Published:** 2013-11-25

**Authors:** Yew Kong Lee, Wah Yun Low, Chirk Jenn Ng

**Affiliations:** 1 Department of Primary Care Medicine, Faculty of Medicine, University of Malaya, Kuala Lumpur, Malaysia; 2 Faculty of Medicine Dean’s Office, Faculty of Medicine, University of Malaya, Kuala Lumpur, Malaysia; Leiden University Medical Centre, The Netherlands

## Abstract

**Background:**

Patient decisions are influenced by their personal values. However, there is a lack of clarity and attention on the concept of patient values in the clinical context despite clear emphasis on patient values in evidence-based medicine and shared decision making. The aim of the study was to explore the concept of patient values in the context of making decisions about insulin initiation among people with type 2 diabetes.

**Methods and Findings:**

We conducted individual in-depth interviews with people with type 2 diabetes who were making decisions about insulin treatment. Participants were selected purposively to achieve maximum variation. A semi-structured topic guide was used to guide the interviews which were audio-recorded and analysed using a thematic approach. We interviewed 21 participants between January 2011 and March 2012. The age range of participants was 28–67 years old. Our sample comprised 9 women and 12 men. Three main themes, ‘treatment-specific values’, ‘life goals and philosophies’, and ‘personal and social background’, emerged from the analysis. The patients reported a variety of insulin-specific values, which were negative and/or positive beliefs about insulin. They framed insulin according to their priorities and philosophies in life. Patients’ decisions were influenced by sociocultural (e.g. religious background) and personal backgrounds (e.g. family situations).

**Conclusions:**

This study highlighted the need for expanding the current concept of patient values in medical decision making. Clinicians should address more than just values related to treatment options. Patient values should include patients’ priorities, life philosophy and their background. Current decision support tools, such as patient decision aids, should consider these new dimensions when clarifying patient values.

## Introduction

Patient decisions are influenced by their personal values; however, there is a lack of clarity and attention on the concept of patient values in the clinical context. This is despite clear emphasis on patient values in evidence-based medicine (EBM) and shared decision making (SDM) [1], [2], [3], [4]. EBM advocates that patients and clinicians make a choice together after considering the best available evidence, the clinician’s experience and the patient’s values [3], [5].

Current definitions of patient values are often vague (e.g. patient values are “the features that matter most to patients [6]”, “the unique preferences, concerns and expectations each patient brings to a clinical encounter and which must be integrated into clinical decisions if they are to serve the patient” [1]) or too narrow. For instance, international standards for patient decision aids narrow the scope of value clarification methods to patient views on physical, psychological and social effects, and the positive and negative features that matter most to patients [7].

To date, most studies on the patient role in shared decision making have focused on measurable patient outcomes, such as more accurate risk assessment [8] or increased patient involvement during consultations [9]. Little research has been conducted on how patients actually choose between options and the patient voice is missing from the conversation [10], [11].

Previous studies have reported that values function as a filter through which patients interpret clinical evidence [12], [13], [14] and make treatment choices [12], [15]. Understanding how values influence patient decision making is particularly relevant to preference-sensitive decisions where there are trade-offs or when there is no one best option. Insulin initiation is one such example of a ‘difficult’ decision which is influenced heavily by patient values [16], [17]. This is particularly important in the context of diabetes which is reaching epidemic proportion and has significant morbidity and mortality [18].

This study used insulin initiation as an exemplar to explore patient values and proposed to create a new model to explain patient values in the context of decision making. It aimed to explore and define patient values because this may help clinicians to understand and address patient concerns and expectations when making decisions.

## Methods

### Ethics Statement

This study received ethics approval from the Medical Research and Ethics Committee, Ministry of Health, Malaysia (Ref No: NMRR-10-1233-7299) and the Medical Ethics Committee, University of Malaya Medical Centre, Kuala Lumpur (MEC Ref No: 841.6).

### Methodological Approach

Due to the lack of literature on values from patients’ perspective, and the exploratory nature of the study, a qualitative study design was chosen [11]. We conducted individual semi-structured in-depth interviews to explore each patient’s values within their experience of insulin initiation. This study formed part of a larger three-year project to develop a decision support tool for clinicians and patients who are making decisions about insulin therapy.

### Conceptual Framework

Our study was developed from the perspective of a SDM model. We used the Ottawa Decision Support Framework (ODSF), an SDM implementation framework, as the conceptual framework within which patient values are nested [19], [20]. The ODSF identifies the decisional needs of patients as values, decisional conflict, knowledge and expectations, support and resources, decision characteristics, and, personal characteristics. Patient values are defined in the ODSF as the “desirability or personal importance of outcomes of options” [20]. We developed a topic guide with 16 questions exploring two main decisional attributes: barriers and facilitators to insulin initiation and barriers and facilitators to decision making; the former focused on patient’s perceptions about insulin itself, while the latter explored the patient’s experience of the decision making process. In order to explore in-depth the topic of values, we then incorporated Schwartz’s theory of values, which is a psychological theory relating to the priority and function of human values [21]. In this theory, the five key attributes of values are: “(1) values are concepts or beliefs; (2) values pertain to desirable end states or behaviors; (3) values transcend specific situations; (4) values guide selection or evaluation of behavior and event; and (5) values are ordered by relative importance” [22]. Table 1 shows the seven questions in our topic guide which explored these five attributes in the context of insulin initiation.

**Table 1:** Semi-structured interview topic guide and corresponding value attribute in Schwartz’s Theory of Values.

Interview questions	Corresponding value attribute in Schwartz’s Theory of Values (if any)
**Part 1: Introduction and rapport building**	
Q1. Can you tell me about your history of diabetes	
**Part 2: Focusing on beliefs about insulin and values**	
Q2. Have you been asked to start insulin? By whom?	
Q3. What has been going through your mind since you were advised to start insulin?	**Values are concepts or beliefs.** We probed the patient’s beliefs about insulin such as negative or positive perceptions, and sources of beliefs.
Q4. Where do you get your ideas/beliefs about insulin from?	
Q5. Is starting insulin a difficult decision for you? Why or why not?	**Values guide selection or evaluation of behaviour and events.** We explored if patients were motivated to start or avoid insulin and their reasons for doing so.
Q6. Are you motivated to start insulin? Why or why not?	
Q7. Have you received any information about starting insulin?	
Q8. What are important priorities to you at this stage of life?	**Values pertain to desirable end states or behaviours.** We explored patients’ life priorities as an operational definition of desirable end states.
Q9. Do these influence your decision to start insulin? If yes, how so? If no, why not?	**Values are ordered by relative importance.** We probed if patients valued some priorities over others, and if priorities had changed over time with different stages in life.
	**Values transcend specific situations.** We explored if non-health related priorities influenced patients’ decisions about insulin.

### Setting

This study was conducted in Malaysia, which is an upper-middle-income, multi-cultural country comprising three main ethnicities (Malay, Chinese and Indian) [23]. Malay is the official language but English is widely spoken in urban areas. Malaysia has a dual healthcare system. The public sector consists of government-subsidized hospitals and health clinics, which serve the majority of the population; the private sector comprises fee-for-service hospitals and clinics. Patients are free to choose where they prefer to receive treatment.

Malaysia has the tenth-highest prevalence rate of diabetes in the world [24], [25] and 70–80% of the Malaysian patients in the primary care setting fail to achieve target HbA1c levels of ≤7.0% [26], [27]. The Malaysian clinical practice guideline recommends insulin initiation in patients with type 2 diabetes mellitus who are poorly controlled despite taking optimal oral glucose-lowering drugs [28]. However, insulin uptake remains poor [24].

### Sampling

Our sample included a range of patients at various stages of decision making. Patients with type 2 diabetes who were still considering insulin or had made a decision about insulin within the past 1 year were included in this study. We decided on this range considering the range of patients’ decision making times is varied for insulin initiation. Unlike one-off medical decisions (such as screening tests or surgery), insulin initiation is a decision that may be considered over a prolonged period of time; patients may change their views about insulin before, during, and after initiation [29]. Clinicians recruited patients whom they had recently advised to start insulin.

Purposive sampling was used whereby we recruited non-randomized participants with specific characteristics in order to achieve maximal variation based on three factors: healthcare setting, patients’ decision about starting insulin, and their ethnicity. To achieve a broad socio-demographic spectrum in the sample, we recruited patients from public and private, as well as rural and urban settings. We included patients who were reluctant to initiating insulin therapy as well as patients who were motivated to initiate insulin therapy. As the interviews progressed, we constantly reviewed the sample characteristics and updated the clinicians on the types of patients we were interested in.

### Data Collection

An interview topic guide was developed based on literature review, conceptual framework and expert opinion (Table 1). The topic guide was pilot-tested and iteratively modified based on themes that emerged during both pilot and subsequent interviews. Both the participant information sheet and topic guide were translated into Malay and Chinese by researchers who were fluent in these languages. Before each interview, participants were given an information sheet and written consent was obtained to participate in the study.

Semi-structured in-depth interviews were conducted with patients in their preferred language (English, Malay or Chinese). Interviews were conducted by three researchers trained in qualitative research methods (YK, CJ, and PY) and each lasted 30–45 minutes. Researchers arranged to interview patients at a time and location of their convenience, including their homes or workplaces if patients were unable to travel due to work commitments or infirmities. Participants were reimbursed for their time and travel. Although the patients were informed that they would be participating in an individual interview, four were accompanied by family members. In such instances, care was taken to avoid having the family members dominate the discussion by consciously focusing questions on the patient.

### Data Analysis

English and Malay interviews were transcribed verbatim while Chinese interviews were translated into English for analysis. Malay interviews were not translated as all researchers were familiar with the language. A thematic analysis approach was used for data analysis, based on Strauss and Corbin’s method of open, axial and selective codes [30]. Three researchers (YK, WY, and CJ) independently coded two interviews line by line to develop an initial list of codes (open coding). A process of constant comparison was employed whereby subsequent interviews were coded using this list and new themes which emerged from new interviews were added to the list upon consultation with the research team. Any discrepancies in the coding process were resolved by discussion during monthly research meetings.

Codes were organised and re-organised into broader categories based on thematic similarities between codes (axial coding). Selective coding was conducted to generate central or core categories based on connecting and consolidating axial codes. All codes were checked by two researchers (YK, CJ) to ensure consistency of coding and consensus on axial and selective codes.

Data collection was stopped when data saturation was reached. Evidence of data saturation was obtained when no new axial or selective codes emerged from the data, showing that the core categories had already been captured. A secondary saturation criterion was based on the saturation of open codes, as there was evidence of repeated coding within the same codes.

Data analysis was facilitated by the use of Nvivo9 software to manage transcripts, themes and quotes, while keeping in mind the context of the quotes within individual interviews.

## Results

### Sample Characteristics

A total of 21 patients were interviewed between January 2011 and February 2012 from five different healthcare locations (one public hospital-based primary care clinic, three public health clinics, and one private clinic). Table 2 details the range of patients interviewed. Although most patients were from an urban setting, they came from diverse socio-economic background. We achieved good variation in our sample in terms of healthcare setting, patients’ decision about starting insulin, and ethnicity. Three core categories of themes emerged: 1) Insulin-specific values, 2) Life goals and philosophies and 3) Socio-cultural values and personal background.

**Table 2:** Characteristics of participants. Values are numbers unless stated otherwise.

Characteristic	Participants (n = 21)
Male	12
Mean (SD) age (years)	55.24 (9.14)
Age range (years)	28–67
**Status of insulin use**	
Not currently on insulin	13
Already using insulin	8
**Healthcare setting**	
University hospital based primary care clinic	7
Public healthcare clinics	8
Private clinic	6
**Language used during interview**	
Malay	9
English	10
Chinese	2
**Ethnicity**	
Malay	6
Chinese	5
Indian	10
**Decision about insulin**	
Keen to start insulin	10
Not keen to start insulin	8
Undecided	1
Not applicable (previous insulin users- gestational diabetes (n = 1) and short-term insulin use(n = 1))	2

### Treatment-specific Values

When making decisions whether or not to start insulin, patients had specific beliefs and feelings about insulin (treatment-specific values). Examples of participants’ perceived advantages and disadvantages of insulin are reported in Table 3.

**Table 3:** Beliefs and feelings about insulin.

Themes	Participant quotes
**BELIEFS ABOUT INSULIN**	
**Positive beliefs about insulin**	
Improve control of diabetes	“To me, I feel that maybe the (oral) drug does not help, then have to use the insulin. I was prepared because I see that my reading, ah, never come down” *F8, female, 57 y.o., private general practice*
Prevent diabetes-related complications	“So I’m thinking, if I’m sixty years old, how long more can I live? Can I put ten more years, can I put twenty years? So why wait till, you know, when my diabetes is very bad and then put full dose of insulin. Try it now and see.” *F4, female, 61 y.o., public hospital-based primary care clinic.*
Minimal side-effects	“Insulin is what our body is producing, you see, rather than all these chemicals going into the body. So it’s just that we take the insulin, it’s easy, direct, no…side effects. I mean, there should be minimal side effects.” F*4, female, 61 y.o., public hospital-based primary care clinic.*
Enable the patient to lead normal lifestyle	“[The doctor] said we give you insulin, means you can eat, no need to control (your diet). You don’t want to eat, or you want to eat, this (insulin) is better. That’s why I said, straightaway said I want it” *M3, male, 63 y.o., public health clinic.*
Convenience of once-daily injections	“[Insulin] is convenient. If you’ve injected in the morning then at night you don’t have to inject” *M12, 61 y.o., private general practice*
Medication adherence is improved	“But if you take insulin every day, you won’t forget. Tablets sometimes you forget. Insulin you know that when you wake up in the morning, you have to inject. Oh, it’s time to eat, it’s time to inject. For tablets, you’re working, working, working and then you have this tablet and that tablet, take half hour after meal, you forget. You go to a restaurant, at that time, you take your tablets, and you need water, right? Ah, you have to look for water. For him (insulin-users) you don’t have to, no need to look for water, just inject insulin.” *F6, female, 58 y.o., public hospital-based clinic*
**Negative beliefs about insulin**	
*Injection-specific beliefs*	
Scarring	“I don’t want to start the insulin. My main concern is the injection and the scar. Everyday injecting, you know, I’m worried it will leave a scar. Because, diabetic people, when you have small injuries, you’ll get black scars, I think my legs have got some. ” *M10, male, 55 y.o., public hospital-based clinic*
Risk of infections	“I’m afraid of, if I start injections tomorrow, will I get any side-effects? Usually, for people with diabetes, when they get a wound, it gets infected, right? Ah, I’ve seen a friend, his leg got cut by a wire, infected and pus-filled.” *M7, male, 67 y.o., public hospital-based clinic*
Easier to forget to take injections	“And then, if they (people who take insulin) missed one day, also it’s a problem. So that’s the reason why I don’t want to take insulin, I’ve been taking medicine for all this while. Medicine is a habit to me, every day I take, I’m reminded to take. Insulin, no, I mean, you might forget.” *M10, male, 55 y.o., public hospital-based clinic*
Interference with current lifestyle	“The way the nurses, the dieticians and the diabeticians and the doctors told me look you must align yourself so they have here 4 meals or 3 meals or whatever and the insulin jabs would correspond to meals. I never take regular meals and the thing is like um… when we have problem with diabetes it's simply because we cannot cope with that huge amount of glucose in our body so human beings physiologically shouldn’t eat big meals you see we only supposed to have small parts throughout the day. But that was what I was trying to do and then the way that they told me is just that…is contrary to what I’ve been doing.” *M1, male, 47 y.o., public hospital-based clinic*
Injection- and needle-phobia	“It’s just that the jabs bothered me at that time. The thing is I don’t like poking myself… that’s normal and the thing is you know like uh… you… doing it 4 times a day you know it's not easy and I mean it was like you have to do it really… I mean sort of like I don’t know you have to have a very good angle to it and then you won’t feel anything and there are some parts that you, there are some places where you cannot just push it through.” *M1, male, 47 y.o., public hospital-based clinic*
	“I’m really afraid of needles. And my daughter told me, how about the needles, right. It’s tiny, you better be careful, if it breaks.” *F3, female, 48 y.o., public health clinic*
Preference for oral tablets or lifestyle intervention	“I feel that I can control my own body. That’s all I think about. When I can’t control (my diabetes), my body doesn’t have enough exercise, that’s the time that I will take insulin. So, now, I have enough exercise, I can control. That’s all.” *M6, male, 56 y.o., public health clinic.*
Social stigma attached to injections	“Will I look like a drug addict? That’s the reason I don’t want to take insulin. It’s just like a drug addict, you know, on the road. They inject themselves, you know, to make them high. This insulin also you have to inject yourself. So you look like a drug addict. I’m not a drug addict, because I only smoke, that’s the only thing I do. So I don’t want to go into the stage where injection, injection, injection.” *M10, male, 55 y.o., public hospital-based clinic*
*Insulin-specific beliefs*	
Unsure about the origin	“I think, quite a number of my friends were not, maybe SPM (high-school) level ah, don’t know that insulin is a natural body made product. They think it’s a very strong medicine, that kind of attitude.” *F4, female, 61 y.o., public hospital-based clinic*
Damaged organs	“I told (my friends) I got to take injections and all that. They err, they said, you inject here, the behind gets spoilt. (Interviewer: Behind? Kidneys?). Yeah, sooner or later its spoilt. My aunties use it, injections. They said kidneys have a lot of problem. That’s why they say, just take oral tablets. Don’t take injections, just eat medication, let go of bad habits, reduce your food and all that.” *M4, male, 53 y.o., public health clinic*
Fear of hypoglycemic events	[Interviewer: So previously, was it your work that caused you to stop insulin?] No, it was the sweat, I have the sweat. So every night, I have to…shivering and wake up. So I was panic, you know. So I stopped it.” *M11, male, 57 y.o., private general practice*
**FEELINGS ABOUT INSULIN**	
**Positive feelings about insulin**	
Normalization	“Insulin is better, I think so, means, I’ll recommend insulin. Because now I see all the people taking insulin, later on, I also take, it’s better.” *M3, male, 63 y.o., public health clinic*
Acceptance	“So I have no choice in that (insulin)…and it’s just that when they found that the levels were not good, that’s when they said it would be better to start on the insulin. Because they gave this very good analogy saying that it is like throwing salt into the sea. You see… when you throw salt into the sea there’s no effect. So that’s the kind of analogy…so I have to change.” *F1, female, 58 y.o., public hospital-based clinic*
**Negative feelings about insulin**	
Severity of diabetes	“My response (to starting insulin) was that my diabetes was not that serious ah. As I said, I will not take it for the time being, I want to observe for a while and see how it goes. [Interviewer: You feel, that if other people take insulin, under what conditions do you think it is important to take insulin?] It is very serious already, when no cure from medicine, then only take this insulin, isn’t it?” *M8, male, 60 y.o., private general practice*
Denial (patient had been advised by doctor to start insulin)	“[Interviewer: So, it was Dr. H who asked you to start insulin, right?] Patient: No, he didn’t, he didn’t. [Interviewer: Oh…sorry.]”. *M11, male, 57 y.o., private general practice*
Frustration or failure	“I think it’s basically attitude change but it’s rather a difficult step *lah*, that transition (to insulin) was difficult. For me, it’s like failing an exam. I tried with so many medications as each time she increases the medications I get depressed. Very sad, ah, it’s getting bad, it’s getting bad.” *F4, female, 61 y.o., public hospital-based clinic*
Feeling punished or threatened	“She (the doctor) say…she scare, she want to scare me. She said, “So high your reading! 10 point something, just now it was like that. 10 point something, you so high, I must put you on insulin all that”. I said, please don’t do that, I say.” *F5, female, 66 y.o., public hospital-based clinic*

#### Beliefs about insulin

The most commonly mentioned advantage was that insulin would help control diabetes and thus prevent diabetes complications. Some thought that insulin would replace oral glucose-lowering tablets (fewer medications) while others believed that insulin had fewer side-effect than tablets. Furthermore, some were reluctant to increase their daily number of oral tablets. One participant had the misperception that insulin was only injected once a week.

However, the majority of participants had negative perceptions about insulin. They expressed doubt over the origin of insulin; concern over insulin side-effects (e.g. hypos); believed that insulin might cause kidney failure and impair pancreatic function. Cost of insulin was also a concern for patients from poor socio-economic backgrounds and private patients whose insulin was subsidised by their employers.

When I am working, the cost (of insulin) can probably be covered. But, when I’m not working? Who wants to cover? Like I say, insulin isn’t bad, it’s good. But, it’s the cost. Cost and for me, how long you want to stick to that kind of medicine. It’s expensive, I know, and that one (insulin) is indeed expensive.

F9, female, 43 years old (y.o.), private general practice.

Moreover, patients had injection-related concerns including: pain, fear of needle, scarring from injections, lifestyle interference, infection at injection sites, forgetting to inject and insulin storage. Some participants were not aware that finer, less-painful needles were available. Two participants were afraid that the needle would break during injections.

#### Feelings about insulin

Participants also reported positive and negative affection about starting insulin. When advised to start insulin, some patients felt that their diabetes was worsening while others denied the need for insulin. They were not confident to self-inject; there was a sense of frustration or personal failure and felt that they were being punished for not controlling their diabetes. Conversely, some had a more positive affection about insulin initiation. They considered insulin initiation as a natural disease progression. They also gained confidence in insulin therapy by discussing with peers who used insulin.

### Life Priorities and Philosophies

When asked what was important in life that might influence their decision making, the participants’ responses could be coded into two categories: life priorities and general philosophies.

### Life Priorities

Life priorities were specific goals in life. Three types of life priorities emerged from the interviews: health, finance and career.

#### Health

Health was a major priority for four participants. Two participants said that health was more important than finance. They said, “It’s OK, we can spend a lot of money. Waste money even, if it’s to look for medicine. We want to look after our body.” (M4, male, 53 y.o., public health clinic) and “Even if I have a lot of money, if we are not healthy, it’s unacceptable” (M7, male, 67 y.o., public hospital-based clinic). One patient said that awareness of risk of diabetes complications “puts you at fear, [you could be that close to] death” (M9, male, 28 y.o., public hospital-based clinic).

#### Career/Employment

Interviews with patients who put priority on career or employment served to illustrate how different patients expressing a similar priority could frame insulin either positively or negatively. For example, one patient viewed insulin positively as he believed it helped him to control blood glucose spikes that had hindered his concentration during work. The other patient viewed insulin negatively as it would interfere with his work schedule.

“Establishing myself in terms of career …my sugar is under control and then I can still hope for the future in terms of careers prospects because I don’t get the sugar spikes anymore, you know” (positive view of insulin).

M9, Male, 28 y.o., public hospital-based clinic.

“I feel good if I go to work…it’s difficult for me to take insulin in the morning, because I have to leave for work at 5 am. We have to think about this as well.” (negative view of insulin).

M6, Male, 57 y.o., public health clinic.

#### Finance

Finance was a priority mentioned by three patients. Insulin-related costs were a concern for them. The need for a self-monitoring blood glucose (SMBG) meter caused one patient to say, “My priority is surely (pause) finance. Doctor A told me to buy the diabetes monitor; she said it’s sold here. The problem is…I can’t afford it. It’s hard being a taxi driver, because taxi rental is fifty ringgit (GBP 10) everyday” (M5, male, 44 y.o., public health clinic).

#### Hierarchy of life priorities

A hierarchy of priorities existed for participants. For example, health was more important than finance. However, priorities were sometimes co-related; one participant reasoned that health was important because it helped to achieve her financial goals.

“I was thinking, like, if I want to save my money, I must take care of my health. Hah, that’s why I go for exercise, you see. Exercise is important. And diet. That’s my concerns.”

F9, female, 43 y.o., private general practice.

### Life Philosophies

Some patients framed insulin according to their life philosophy. In contrast to life priorities, which are concrete goals that are important to the patient, life philosophies are related to patients’ worldviews and ethical beliefs about what are morally desirable.

#### Avoiding suffering

Avoiding suffering was a recurring theme. One participant stated that his view on life was to “die happy” and that he would consider taking insulin because he didn’t want to “suffer and die” (M3, male, 63 y.o., public health clinic).

Another said that “If my suffer(ing)s are very major, I’m going to be dependent on anybody, I might as well go kill myself, instead of living with all the suffering and whatever nonsense that’s going on.” (M10, male, 55 y.o., public hospital-based clinic).

Another participant associated suffering with death by saying she prayed that “Please help me, I don’t want to suffer pain. If I live, just let me live normally. When I die, don’t let me suffer pain or anything, don’t let me die that way” (F6, female, 56 y.o., public hospital-based clinic).

#### Fatalism

Some participants refused insulin treatment as they felt that everyone was fated to die one way or another.

“About dying, I’m not worried about it because these things they come naturally. Die means you die, no helping it. You inject until he dies, also die in the end, it’s like that. So there is nothing to worry about.”

M8, male, 60 y.o., private general practice.

#### Not being a burden

Not burdening others was the most important philosophy for one lady. She explained that “I don’t want to be a burden to anybody and as well as to myself. I want to be independent, and a helpful person. That’s the thing that’s making me agree to insulin” (F4, 61 y.o., public hospital-based clinic).

### Socio-cultural Values and Personal Background

Patients’ decision to start insulin was also shaped by their larger social environment, belief system (e.g. religion), and personal background (e.g. family context).

#### Religion

Religious values were a factor that influenced patients’ views about insulin. Four participants were concerned that the use of insulin might conflict with their religious beliefs. A Muslim patient was concerned about the purity (‘halal’) of insulin and needed assurance from a Muslim clinician. A Hindu patient illustrated how insulin injections could potentially desecrate holy sites as religious rules forbade blood being spilt inside temples.

I wouldn’t like to be in a (Hindu) temple, take out my needle and jab, I don’t think it’s nice. Because that’s supposed to be a spiritual, clean place. So my son-in-law was, like, arguing with me that day and said the blood doesn’t come out. In a spiritual place, blood shouldn’t come out as if it will fall on the floor of the temple, it’s a very big (pause) sin.

F4, female, 61 y.o., public hospital-based primary care clinic.

#### Personal and family background

The following example illustrates how a 66 year-old woman’s family context influenced her decision to avoid insulin. For this patient, her insulin-specific belief was the perception that insulin was expensive.

“I feel I want to save money. Insulin is expensive; I don’t want to take it.”

F5, female, 66 y.o., public hospital-based clinic.

When asked what was important to her in life, she said that her life priority was on work. This was related to her view that her children were unable to support her.

“I am mostly thinking about work. My son in law, children…how much money can they give? My daughter has her own family, my son also has his own family.”

Finally, when probed why work was prioritised, it emerged that this was due to her tight financial situation. She had to work to support her family and provide for her children’s studies after her husband became ill.

“I suffer a lot. My husband retired at fifty-five. Because the doctor asked him to stop working, that time he has a heart problem. That’s why every cent I earned, I give it to my son and daughter to study.”

## Discussion

This study aimed to explore patient values and what role it plays when making a health decision. The study identified a range of patients’ positive and negative perceptions of insulin as well as life priorities and philosophies that influenced patients’ decision making. Through analysis of patient narratives, we illustrate how patients’ personal background also influenced their decision about insulin. The study expands the current definition of patient values as treatment preferences to cover a broader dimension including personal life goals and philosophies.

The strength of this study is that the theoretical framework was drawn from a social science theory of human values. By broadening our scope of values to those outside of healthcare, we illustrate how priorities such as career achievement and ethical convictions are influential in patient decision making. Thus, the complex interactions between treatment-specific beliefs, goals and contextual background that emerged from the data are more holistic and, we believe, provide a more accurate representation of actual patient values.

The limitations of our study are that the specific themes from this study may not be transferable to other conditions. Patient values are shaped by local culture and norms. Therefore, priorities and philosophies identified in this sample of patients may not be similar to patients elsewhere.

The first category of values comprised of beliefs and feelings about insulin. These influenced patients’ view of insulin as being either positive or negative. Firstly, patients have a set of cognitive beliefs about the perceived advantages and disadvantages of insulin (refer to Table 3). Not all of these beliefs are correct; patients also reported misperceptions about insulin. Besides cognitive concepts of insulin, patients also expressed an affective concept of insulin i.e. how insulin made them feel. Denial, punishment or lack of self-efficacy would influence patients to view insulin negatively.

Our study reports that patients in Asia share similar beliefs about insulin as those in the west, such as the fear of injections [31], [32], inconvenience when using insulin [17], [33], fear that insulin will cause organ damage [32], [34], and feeling a sense of failure or punishment [32], [35]. However, while most studies only highlight medically-related barriers concerning the efficacy and side-effects of insulin [36], our study underlines the importance of exploring non-medical beliefs as potential barriers during insulin initiation. Some examples in our study include religious beliefs about blood, and patients’ fear of social stigma from associating drug use with injection scars. Such socio-cultural and religious concerns may be factors for higher insulin refusal rates in Asian populations (42–52%) [36], [37], [38] compared to the west.

Besides insulin-specific beliefs, other non-health beliefs also influenced patients’ decisions. Patients would consider if insulin agreed with their system of life goals and philosophies. In other words, the choice about insulin was interpreted according to the patient’s worldview. Previous literature has highlighted different types of patient values that should be considered when making a healthcare decision [39]. Schwartz et al have reported that from a list of seven ‘life goals’ (family, wealth, job, education, health/fitness, travel, and personal fulfilment), participants were significantly more willing to trade off achieving family goals for health or life years compared to other goals [40]. Such value typologies however face the limitation of being either conceptual or hypothetical. Our study adds to the literature by reporting on patients actually used values when considering insulin. Besides weighing the pros and cons of insulin from a medical perspective, patients also viewed if insulin would be congruent with their worldview, which includes their life goals and philosophies.

### Implications for Practice

Currently, patient education remains the cornerstone of counselling patients who are resistant to insulin [41], [42], [43]. The majority of the interventions focus on motivating patients to start insulin by changing their perceptions about insulin (e.g. normalization of insulin) and challenging negative perceptions about insulin use. There is little discussion about decision support and whether the treatment agrees with patients’ values. One reason for this is the assumption that both HCPs and patients share similar values [39]. This study shows that patient values may not be congruent with health-seeking goals. Thus, besides addressing patients’ negative perceptions, HCPs must also explore patients’ underlying value motivations [44].

**Figure 1:**
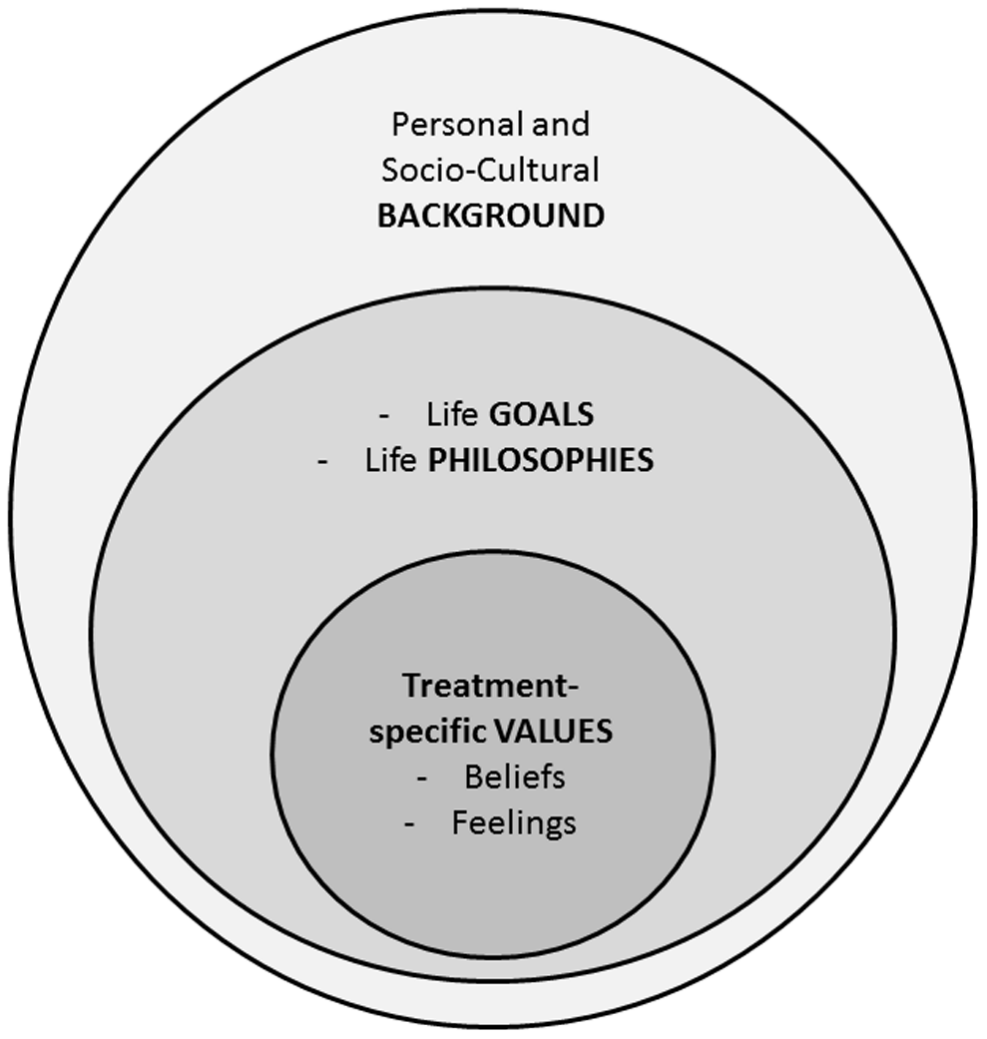
A conceptual model of patient values.

From our analysis, patient values comprise three key categories: treatment-specific values; life priorities and philosophies; and socio-cultural and personal background. In Figure 1 we propose a conceptual model whereby these components form the content of the model and are arranged in three layers. The need to elicit patient values in medical decision making arises within the context of a specific medical decision. As such, the arrangement of the layers in the model was based on how closely related the value categories were to the medical decision being discussed. Whereas the first layer (treatment-specific values) are beliefs that are specific to the treatment (e.g. “I am afraid of insulin injections because they are painful.”), the second (life priorities and philosophies) and third (socio-cultural and personal background ) layers are trans-situational, meaning that they are applied to other areas besides health. The second layer is the patient’s personal, individual beliefs (which may also include health as a priority) while the third layer comprises cultural and contextual influences. This model expands on the current scope of patient values in EBM [1], [12] and SDM [5] to also include life priorities and philosophies (or a patient’s worldview). In the centre are treatment-specific beliefs which depend on the medical context, while layers further from the centre are more deep-seated and trans-situational, and more importantly, also influence the treatment choice.

Recent literature has expressed the need to consider the broader communicative and relational contexts when practicing SDM [45]. When supporting patients in making decisions, clinicians need to address more than just beliefs and feelings about the treatment options. A deeper understanding of patients’ life priorities and background are essential, particularly when making decisions about treatments. From our study, we suggest that assessing these values involves competencies in eliciting and analysing patient narratives [46], [47], [48], [49]. Understanding patient narratives is especially important for long-term care of chronic diseases which are heavily influenced by factors such as prior and current life experiences, resources, and explanatory models of illness [50].

Further research needs to be done on a number of aspects. Firstly, how generalisable is the proposed conceptual model of patient values? More studies should be conducted in different healthcare decisions, locations and cultures. Secondly, would an intervention targeting goals and philosophies be more effective than management programmes focusing on improving patient perceptions about insulin? One example would be value self-confrontation, [51] where a patient with poor glycaemic control could be shown how their set of values differs from that of patients with good glycaemic control.

## Conclusions

In this paper, we introduce a comprehensive model of patient values based on actual patient perspectives. This model fits well with the practice of EBM and SDM by helping clinicians to understand how patients also consider other non-health values when making a treatment decision. Further study needs to be done to explore the applicability of this model in other contexts.
